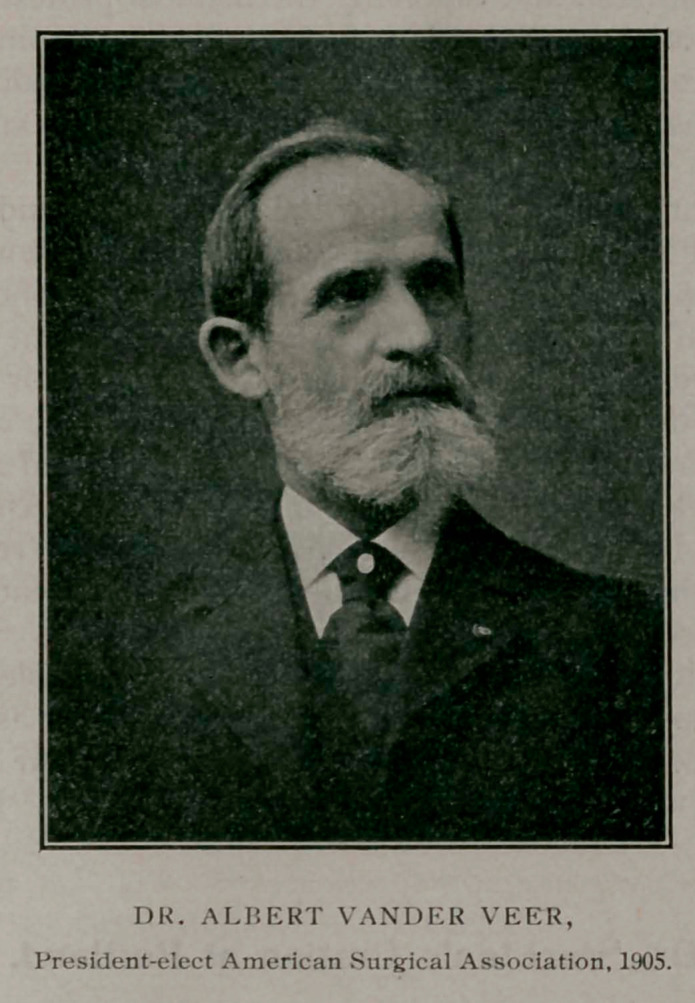# The American Surgical Association

**Published:** 1905-08

**Authors:** 


### The American Surgical Association.

THIS distinguished association held its twenty-sixth annual meeting at San Francisco, July 5-7, 1905, under the presidency of Dr. George Ben Johnston, of Richmond. The program was varied and interesting and included papers by Drs. A. Vander Veer, George R. Fowler and Roswell Park, of the state of New York. We publish elsewhere an abstract of Dr. Park's paper prepared by himself and specially contributed to this journal.

Dr. Johnston chose for the subject of his presidential address, John Peter Mettauer, one of the most eminent surgeons of his day. This biographical sketch embraced an account of Mettauer's surgical career and detailed many of his eccentricities, —one of which was the wearing of an absurdly tall hat at all times and on all occasions.

The association elected Dr. A. Vander Veer, of Albany, to be president for the current association year. Dr. Vander Veer is one of the most eminent surgeons of the day, is Dean of the Albany Medical College and is professor of didactic, abdominal and clinical surgery at that institution. Dr. Vander Veer's contributions to the literature of surgery are many, varied and useful, covering almost every important surgical question that has been discussed during the past twenty-five years. He is a past president of the Medical Society of the State of New York and during several years has served as a regent of the University of the State of New York. Dr. Vander Veer is in the prime of a ripe manhood and is doing some of the best surgical work at the present time. He is one of the earlier Fellows, and the associa-

DR. ALBERT VANDER VEER,

President-elect American Surgical Association, 1905.

tion in his election has conferred upon itself an all too tardy honor.

## Figures and Tables

**Figure f1:**